# Genistein and exercise modulated lipid peroxidation and improved steatohepatitis in ovariectomized rats

**DOI:** 10.1186/s12906-020-02962-z

**Published:** 2020-06-01

**Authors:** Namthip Witayavanitkul, Duangporn Werawatganon, Maneerat Chayanupatkul, Naruemon Klaikeaw, Sompol Sanguanrungsirikul, Prasong Siriviriyakul

**Affiliations:** 1 Alternative and Complementary Medicine for Gastrointestinal and Liver Diseases Research Unit, Department of Physiology, Faculty of Medicine, Chulalongkorn University, Bangkok, 10330 Thailand; 2 Department of Pathology, Faculty of Medicine, Chulalongkorn University, Bangkok, Thailand

**Keywords:** Genistein, Exercise, Nonalcoholic steatohepatitis, Ovariectomized, Estrogen deficiency

## Abstract

**Background:**

The prevalence of nonalcoholic steatohepatitis (NASH) in menopausal women is increasing, but current treatments have not been proven effective. The objective of this study was to investigate the treatment effects of genistein and running exercise in ovariectomized (OVX) rats with NASH.

**Methods:**

Thirty-six female Sprague-Dawley rats were divided into 6 groups, control; OVX with standard diet; OVX with high fat and high fructose (HFHF) diet for 4 weeks; OVX with HFHF and genistein treatment (16 mg/kg BW/day) for 5 weeks (OVX + HFHF+GEN); OVX with HFHF and moderate intensity exercise for 5 weeks (OVX + HFHF+EX); OVX with HFHF and combined treatments (OVX + HFHF+GEN + EX). Serum interleukin-6 (IL-6) levels, hepatic free fatty acid (FFA), hepatic glutathione (GSH), and hepatic malondialdehyde (MDA) levels were measured. Liver histology was examined to determine NASH severity.

**Results:**

OVX + HFHF group had the highest levels of hepatic FFA compared with OVX and control groups (5.92 ± 0.84 vs. 0.37 ± 0.01 vs. 0.42 ± 0.04 nmol/mg protein, respectively, *p* < 0.01). Serum IL-6 levels were significantly elevated in both OVX and OVX + HFHF groups as compared with controls (112.13 ± 6.50 vs. 121.47 ± 3.96 vs. 86.13 ± 2.40 pg/mL, respectively, *p* < 0.01). In OVX + HFHF group, hepatic MDA levels were higher, while GSH levels were lower than in OVX and control groups (MDA; 0.98 ± 0.04 vs. 0.82 ± 0.02 vs. 0.78 ± 0.03 nmol/mg protein, and GSH; 46.01 ± 0.91 vs. 55.21 ± 1.40 vs. 57.94 ± 0.32, respectively; *p* < 0.01 for both). Comparing with OVX + HFHF group, rats that received genistein, exercise and combined treatments demonstrated an improvement in liver histopathology, decreased levels of hepatic FFA (1.44 ± 0.21 vs. 0.45 ± 0.04 vs. 0.49 ± 0.05 nmol/mg protein, respectively*, p* < 0.01), serum IL-6 (82.80 ± 2.07 vs. 83.47 ± 2.81 vs. 94.13 ± 1.61 pg/mL, respectively, *p* < 0.01), and hepatic MDA (0.80 ± 0.03 vs. 0.76 ± 0.02 vs. 0.76 ± 0.03 nmol/mg protein, respectively, *p* < 0.01).

**Conclusions:**

Genistein and moderate intensity exercise were effective in reducing the severity of NASH in OVX rats through the reduction in liver inflammation, oxidative stress and liver fat contents.

## Background

The spectrum of nonalcoholic fatty liver disease (NAFLD) comprises simple steatosis, nonalcoholic steatohepatitis (NASH), progressive fibrosis, cirrhosis and in some cases hepatocellular carcinoma. NASH is defined histologically by the presence of steatosis, hepatocyte ballooning, and lobular inflammation [1]. The derangement in lipid and glucose metabolism is a common occurrence in NAFLD. High-calorie diet and high fructose consumption are associated with increased severity in patients with NASH [2]. In addition, excessive energy intake and fat accumulation in the liver can induce oxidative stress and hence severe lipid peroxidation [3].

The prevalence of NAFLD increases in post-menopausal women as compared with pre-menopausal women suggestive of a protective effect of estrogen on NAFLD [4]. Receiving more than 6 months of hormone replacement therapy has been shown to decrease the frequency of NAFLD in post-menopausal women by 34% [5]. Despite its beneficial effects in improving NASH pathology, long term use of hormone replacement therapy increases the risk of breast, ovarian and endometrial cancers. An alternative medicine that can improve NAFLD in the settings of estrogen deficiency without higher risks of cancers is therefore an attractive option.

Genistein (4′, 5, 7-trihydroxyisoflavone, supplement Figure 1), a phytoestrogen which can be found in soybean products, has been shown to prevent lipid accumulation [6]. Moreover, genistein is not associated with increased prevalence of breast cancer [7]. A previous animal study suggested that genistein (16 mg/kg) improved histopathology of NASH by upregulating hepatic PPARγ and reducing oxidative stress markers and inflammatory cytokine in ovariectomized (OVX) rats fed with high fat high fructose (HFHF) diet [8]. Another animal study similarly showed that genistein reduced %NFκB-positive cells and hepatic free fatty acid levels and improved liver histology in both rats with intact ovary and post-ovariectomy fed with HFHF diet [9]. Using specific-pathogen-free male Sprague Dawley rats fed with high fat diet as a NASH model, Yin and colleagues found that high dose genistein decreased NASH activity score, hepatic triglyceride levels, insulin resistance, plasma endotoxin levels and hepatic toll-like receptor-4 expression [10].

Exercise has been shown to improve NASH and metabolic profiles in both animal and human studies [11]. Aerobic exercise in menopausal women improved lipid profiles after 24 weeks of training as compared with a control group [12]. In an animal model of high fat diet induced NASH, moderate intensity exercise training improved histological features of NASH with reduction in markers of hepatic stellate cell activation and extracellular matrix remodeling [13]. Furthermore, combination of exercise and isoflavone for 5 weeks could reduce plasma triglyceride (TG) in male rats [14]. However, it remains unclear whether combination treatment with genistein and running exercise has an additive effect on attenuating NASH in OVX rats fed with HFHF diets compared with either treatment alone. The present study aimed to determine the beneficial effects of genistein, running exercise and combined therapy on steatosis, inflammation and oxidative stress in OVX rats with NASH induced by HFHF diet. We used OVX female rats as a model for post-menopausal women.

## Methods

### Animal preparation

Eight-week-old female Sprague-Dawley rats weighing 200–220 g from the Nomura Siam International Co., Ltd. Bangkok, Thailand were used. The protocol was approved by the Animal Care and Use Committee at the Faculty of Medicine, Chulalongkorn University (IRB No. 018/2561). All animals were kept at the Animal Center, Faculty of Medicine, Chulalongkorn University under strictly hygienic conventional system in a controlled temperature room at 25 ± 1 °C with a normal 12 h light-12 h dark cycle. All rats had free access to purified drinking water. Each group of rats (described in the following paragraph) was housed in a separate stainless steel cage with solid bottom and open top. After one week of acclimatization to the new environment, rats were randomized to the control or OVX groups. Bilateral ovariectomy was performed in OVX groups using double dorsolateral approaches [15]. In brief, after the animal was anesthetized with intraperitoneal injection of sodium thiopental, skin incision was performed and muscle layers were cut. First, both ovaries were identified. Distal uterine horns were then ligated and both ovaries were removed. After ovariectomy, the surgical wound was closed layer by layer. OVX rats were individually caged during a 2-week recovery period. The presence of anestrous stage on vaginal smear at two weeks after the operation was used to confirm the completion of ovariectomy [16].

### Experimental design

Thirty-six female Sprague-Dawley rats were randomly divided into 6 groups, (1) control rats fed with standard diet (2) OVX rats fed with standard diet (OVX), (3) OVX with HFHF diet for 4 weeks (OVX + HFHF), (4) OVX with HFHF and genistein treatment (16 mg/kg BW, once daily) for 5 weeks (OVX + HFHF+GEN), (5) OVX with HFHF and moderate intensity exercise (running 80%VO_2_max, 3 times/week) for 5 weeks (OVX + HFHF+EX), and (6) OVX with HFHF and combined treatments (OVX + HFHF+GEN + EX). The number of animals in each group was calculated with G Power program using values (mean and SD) of hepatic catalase activity in an article from Yoon GE et al. [14] with α = 0.05, power (1-β) = 0.8.

Running protocol was as follows. All rats in the exercise group performed treadmill running five days per week for five weeks. Exercise period progressed from 10 min to 30 min by adding five minutes per week. Speed was maintained at 15 m/minute for the first three weeks then increased to 20 m/minute for the remaining two weeks. The distance run during the first week was 0.15 km/day, the second week was 0.225 km/day, the third week was 0.3 km/day, the fourth week was 0.5 km/day and the fifth week was 0.6 km/day. This protocol was considered a moderate intensity exercise based on results from prior studies [17, 18].

At the end point of the experiment, animals were euthanized using overdose sodium thiopental injection intraperitoneally (dose > 50 mg/kg) after an 8-h fast. Livers were surgically removed, weighed, and cut into several pieces. Half of liver specimens were immediately frozen in liquid nitrogen and stored at − 80 °C until further analyses (Oil Red O stain, and MDA, hepatic fatty acid and GSH measurement). The remaining liver specimens were fixed in 10% formaldehyde for histopathological examination. Blood samples were obtained through cardiac puncture. Serum was then separated by centrifuging the blood at 2000 rpm (r.p.m.) for 20 min at 4 °C. Serum samples were stored at − 80 °C until further analysis. Serum IL-6 levels, hepatic contents of free fatty acid (FFA), gluthatione (GSH), and malondialdehyde (MDA) were measured. Liver histology was examined to determine NASH severity. All procedures were performed at the Alternative and Complementary Medicine for Gastrointestinal and Liver Diseases Research Unit, Chulalongkorn University. The primary outcome was to determine the effects of genistein, moderate intensity exercise and combined treatment on liver histology in rats with NASH. Secondary outcomes included changes in liver fat content and inflammatory and oxidative stress markers in NASH and treatment groups.

The HFHF diet used in this study was modified from Pickens MK formula [19] and contained 55% fat (from palm oil), 35% carbohydrate (consisted of 20% fructose and 80% starch), and 10% protein from albumin. Standard diet contained 7% fat, 47% carbohydrate, and 27% protein (Perfect companion group Co., Ltd., Thailand). Rats had free access to food ad libitum.

Genistein powder at the dose of 16 mg/kg body weight (Cayman Chemical Company, USA), was dissolved in 0.1% DMSO prior to administration to each rat by oral gavage once daily in the morning for 5 weeks.

Moderate intensity exercise protocol was a treadmill running at 80%VO_2_max 3 times a week for 5 weeks as described previously [14]. The speed and duration of the treadmill exercise were gradually increased until the animals could maintain a running speed of 18–20 m/min for 30 min/day on a 0% incline.

### Hepatic free fatty acid measurement

Liver lipid was extracted with a lipid extraction kit (BioVision, Inc., CA, USA) according to the manufacturer’s instructions then suspended in 50 μl of lipid suspension buffer and sonicated for 15–20 min at 37^o^ C. The lipid was used to quantify the amount of FFA by colorimetric assays (BioVision, Inc., CA, USA). Tissue protein concentration was determined using a Pierce BCA Protein Assay Kit (Thermo Scientific, USA). Results were expressed in nmol per mg of hepatic tissue protein.

### Fat droplet measurement

Liver specimens were frozen, sliced with cryostat, and stained with Oil Red O staining. The slides were examined under light microscopy with 400x magnification to detect fat droplets. The amount and individual size of the lipid droplets from three sections of each rat were analyzed using ImageJ program and presented as a percentage of surface area that were stained red [20].

### Serum IL-6 measurement

Enzyme-linked immunosorbent assay (ELISA) kit was used to measure the serum levels of IL-6 by strictly following the manufacturer’s instructions (R&D Systems, Inc. USA). The absorbance was read at 450 nm.

### Hepatic MDA and GSH measurements

Lipid peroxidation was determined by measuring MDA levels in liver tissue using thiobarbituric acid reaction (TBARS Assay Kit, Cayman Chemical Company, USA). Briefly, liver was homogenized and sonicated on ice for 15 s. Supernatants were obtained after centrifugation at 1600 x g for 10 min at 4 °C. The absorbance of the supernatant fraction was read at 530 nm and results were expressed in nmol per mg of hepatic tissue protein.

To determine hepatic GSH, tissue was homogenized and assayed using a Glutathione Assay Kit (Cayman Chemical Company, USA). Briefly, Liver tissue was washed and homogenized before being centrifuged to obtain the supernatants which were then deproteinated. The sulfhydryl group of glutathione reacts with DTNB to form TNB of which the absorbance was measured at 405 nm. The results were multiplied by sample dilution of deproteination and expressed in micro molar (μM).

### Histological evaluation

Formalin-fixed, paraffin-embedded liver specimens were stained with haematoxylin and eosin (H&E) staining for histological evaluation. Pathologists, who were blinded to the experimental groups, graded NASH severity according to Brunt’s criteria which was based on a point system for 3 lesions: steatosis (0–3), lobular inflammation (0–3), and hepatic ballooning (0–2) [21]. NAFLD Activity Score (NAS) is a sum of scores in each category, thus ranging from 0 to 8 [22].

### Statistical analysis

Data from all animals (*n* = 36) were included in the analyses. Continuous variables were compared using one-way analysis of variance (ANOVA) followed by Tukey’s multiple comparison test. Results were presented as mean ± standard error of the mean (SEM), and *p* value of less than 0.05 was considered statistically significant. All analyses were performed using SPSS for Windows version 17.0 (SPSS, Inc., Chicago, IL, USA).

## Results

### Effects of genistein, exercise, and combined treatment on body weight changes

At the beginning of the experiment, the body weight of rats in each group were not different (*p* = 0.988). At the end of the experiment, the body weight of OVX rats markedly increased compared with control group (∆Body weight 165.67 ± 5.51 vs. 78.13 ± 4.76, *p* < 0.01). A significant reduction in the body weight was observed in the all groups of OVX rats fed with HFHF diet compared with rats fed with standard diet. The body weight changes after treatment with genistein, running exercise, or combining genistein and exercise were not significantly different among treatment groups compared with OVX rats fed with HFHF diet.

### Alterations in gross appearance of the liver and liver index

**Fig. 1:**
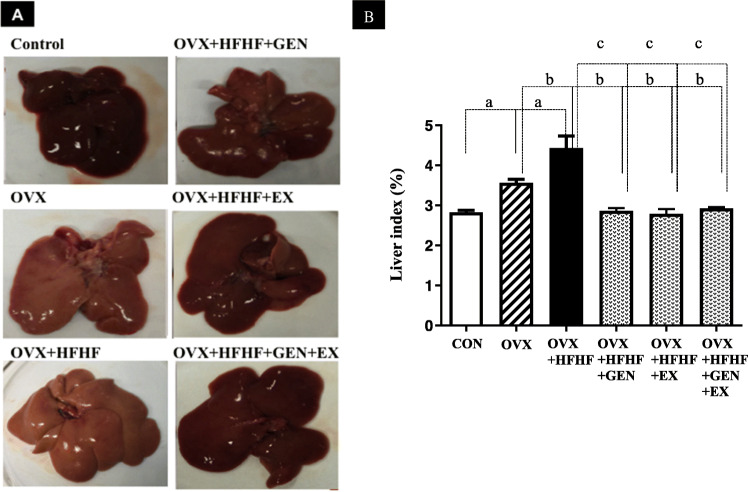
Effects of genistein, exercise, and combined treatment on liver appearance and liver indices in each group

Gross examination of the liver was performed and each liver was weighed. The liver of OVX and OVX + HFHF rats showed yellowish discoloration as compared to normal liver appearance in a control group. Liver indices, the ratio between liver weight and body weight, were significantly higher in both OVX and OVX + HFHF groups compared with a control group (3.57 ± 0.09% vs. 4.43 ± 0.30% vs. 2.82 ± 0.06%, respectively; *p* < 0.01). Treatment with genistein, exercise or in combination could normalize liver appearance (Fig. 1a) and reduce liver indices to the level of a control group (2.86 ± 0.07 vs. 2.79 ± 0.12 vs. 2.93 ± 0.03, respectively; *p* < 0.01) (Fig. 1b).

### Effects of genistein, exercise, and combined treatment on histopathological changes

**Fig. 2:**
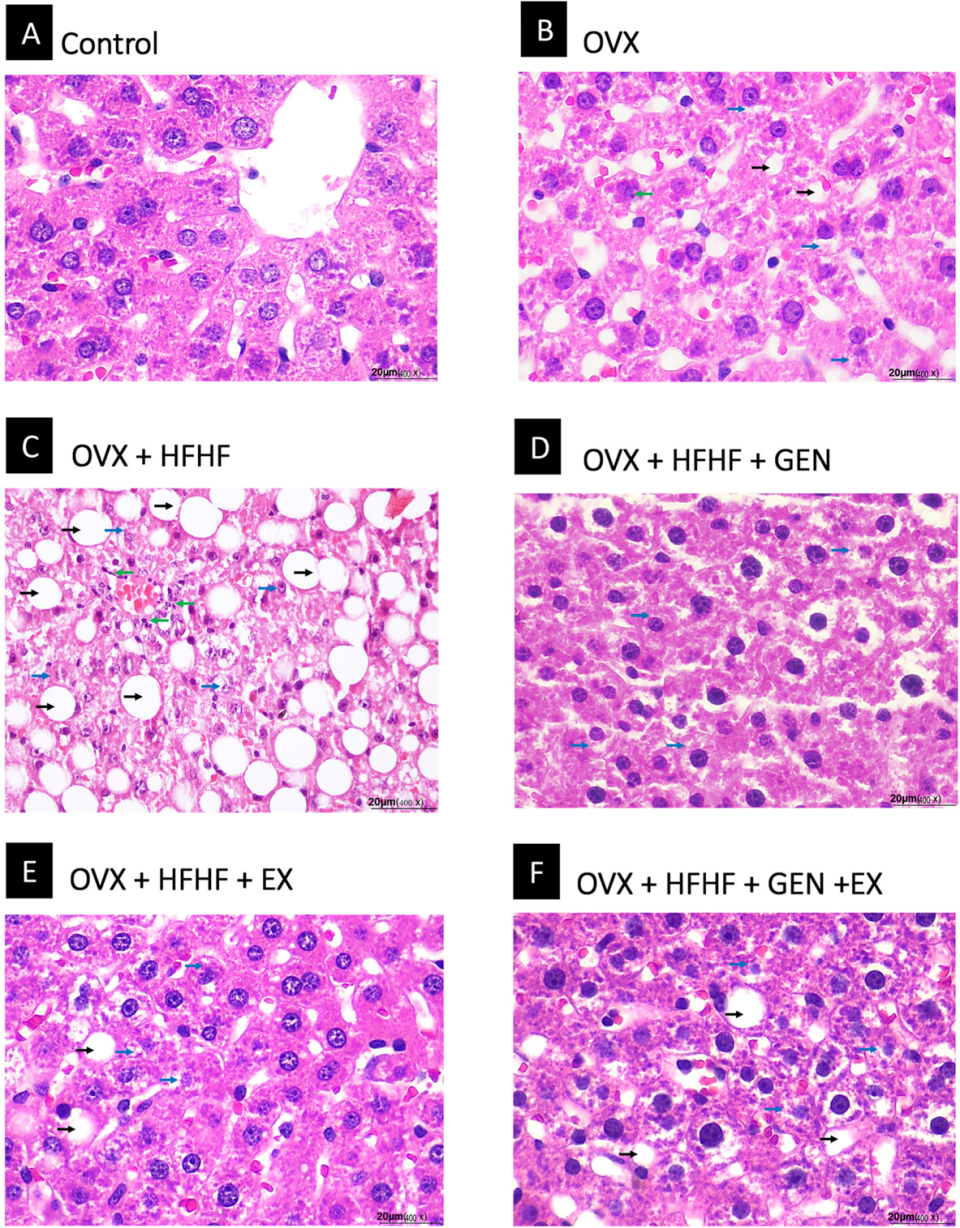
Effects of genistein, exercise, and combined treatment on NASH pathology

**Fig. 3:**
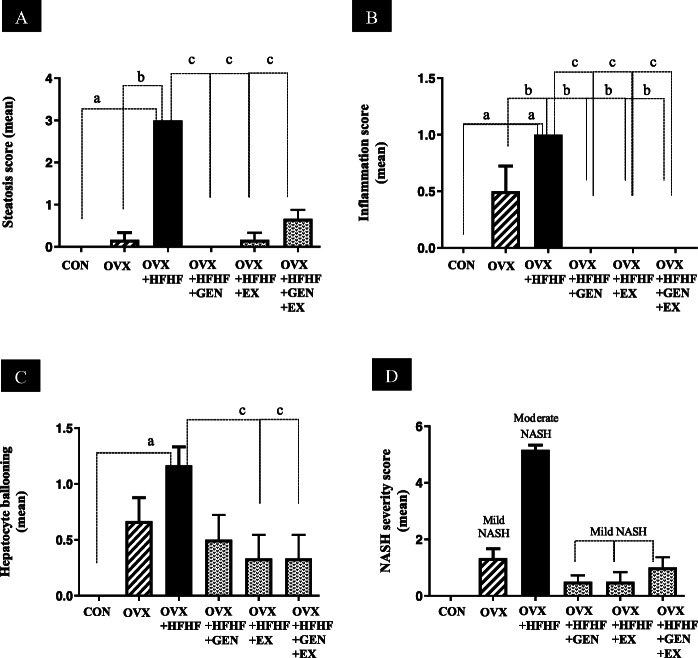
Effects of genistein, exercise, and combined treatment on each histologic feature of NASH and overall NAS score

Figure 2 are representative images of liver histology in each group. The severity of steatosis, lobular inflammation and hepatocyte ballooning and the composite scores of each group are presented in Table 1 and Fig. 3a-c. Mild steatosis, inflammation and hepatocyte ballooning were seen OVX rats, while OVX + HFHF ones exhibited more severe changes in liver histology. Albeit not normalized, treatment with genistein, moderate intensity exercise and combined treatment could reduce the severity of NAFLD in all 3 aspects.

**Table 1:** The summary of steatosis, inflammation and hepatocyte ballooning scores on liver histology in each groups

Group	n	Steatosis				Inflammation				Hepatocyte ballooning		
		0	1	2	3	0	1	2	3	0	1	2
Control	6	6	–	–	–	6	–	–	–	6	–	–
OVX	6	5	1	–	–	3	3	–	–	2	4	–
OVX + HFHF	6	–	–	–	6	–	6	–	–	–	5	1
OVX + HFHF+GEN	6	6	–	–	–	6	–	–	–	3	3	–
OVX + HFHF+EX	6	5	1	–	–	6	–	–	–	4	2	–
OVX + HFHF+GEN + EX	6	2	4	–	–	6	–	–	–	4	2	–

Data are expressed as the number of rats in each of histology grading score. Steatosis grade; 0 = < 5%, 1 = < 33%, 2 = 33–66%, 3 = > 66%. Inflammation grade; 0 = normal, 1 = mild, 2 = moderate, 3 = severe. Hepatocyte ballooning grade; 0 = no ballooning, 1 = few balloon cells, 2 = many balloon cells

### Effects of genistein, exercise, and combined treatment on lipid accumulation in the liver

**Fig. 4:**
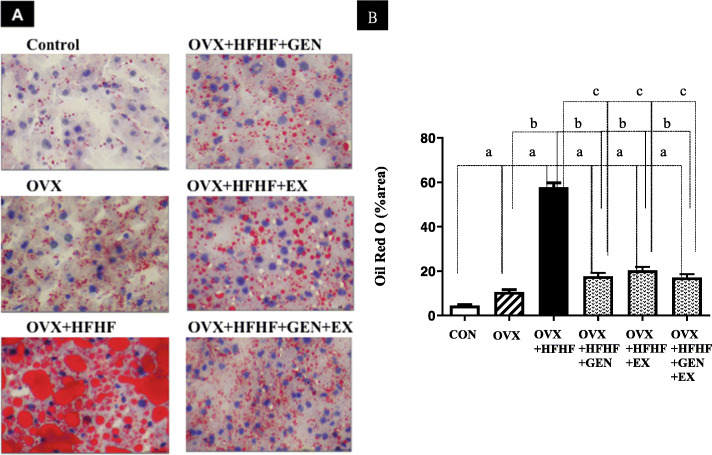
The degree of hepatic steatosis in each experimental group

Figure 4a demonstrated the degree of fat accumulation in the liver of each group using Oil Red O staining. The percentage of positive staining area increased significantly in OVX and OVX + HFHF diet groups as compared with a control group (10.66 ± 1.06% vs. 57.80 ± 1.94% vs. 4.52 ± 0.37%, respectively; *p* < 0.01) (Fig. 4b).

**Fig. 5:**
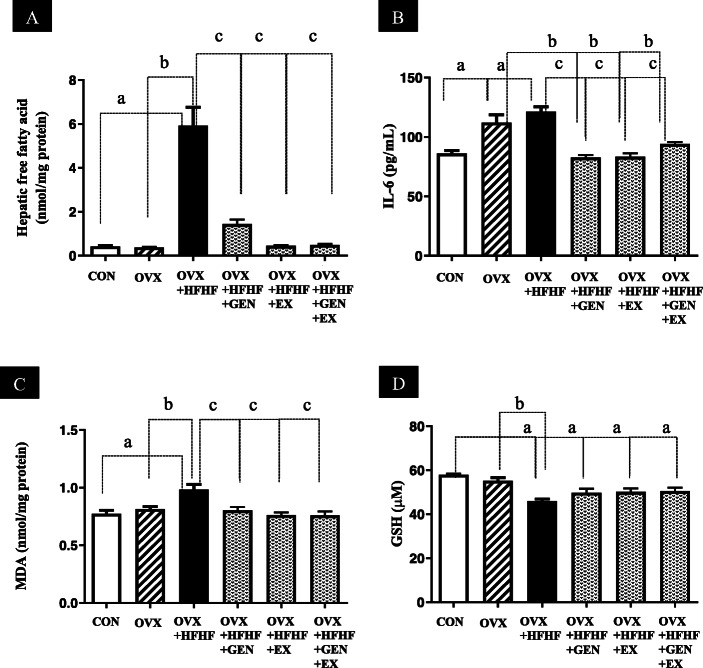
Effects of genistein, exercise, and combined treatment on cytokine levels and oxidative stress markers

Hepatic content of free fatty acid (FFA) in the OVX + HFHF group was significantly higher than in OVX and control groups (5.92 ± 0.84 vs. 0.37 ± 0.01 vs. 0.42 ± 0.04 nmol/mg protein, respectively; *p* < 0.01). After treatment with genistein, exercise or combined treatment, FFA levels in the liver decreased significantly compared with the OVX + HFHF group (1.44 ± 0.21 vs. 0.45 ± 0.04 vs. 0.49 ± 0.05 vs. 5.92 ± 0.84 nmol/mg protein respectively; *p* < 0.01). (Fig. 5a).

### Effects of genistein, exercise, and combined treatment on cytokine levels and oxidative stress markers

As shown in Fig. 5b, serum levels of interleukin-6 (IL-6) were higher in both OVX and OVX + HFHF groups than in a control group (112.13 ± 6.50 vs. 121.47 ± 3.96 vs. 86.13 ± 2.40 pg/mL, respectively; *p* < 0.01). After treatment with genistein, exercise and combined therapy, IL-6 levels decreased to the levels of a control group (82.80 ± 2.07 vs. 83.47 ± 2.81 vs. 94.13 ± 1.61 pg/mL, respectively).

OVX + HFHF group had significantly higher hepatic levels of MDA (Fig. 5c) and lower GSH (Fig. 5d) in comparison to OVX and control groups (MDA; 0.98 ± 0.04 vs. 0.82 ± 0.02 vs. 0.78 ± 0.03 nmol/mg protein, and GSH; 46.01 ± 0.91 vs. 55.21 ± 1.40 vs. 57.94 ± 0.32, respectively; *p* < 0.01 for both). Hepatic MDA levels in genistein, exercise and combined treatment groups closely resembled those of control group (0.80 ± 0.03 vs. 0.76 ± 0.02 vs. 0.76 ± 0.03 nmol/mg protein, respectively *p* < 0.01). On the contrary, genistein, exercise and combined treatment could not restore hepatic GSH back to normal levels.

## Discussion

Estrogen deficiency in post-menopausal women has been shown to be associated with visceral fat accumulation, insulin resistance and NAFLD [23]. An animal model showed that estrogen when bound to liver estrogen receptor-α (ESR1) could inhibit hepatic gluconeogenic genes such as phosphoenolpyruvate carboxykinase 1 (Pck-1) and glucose 6-phosphatase (G6Pase) and decrease hepatic lipogenesis through a down-regulation of fatty acid synthase (Fas) and acetyl-CoA carboxylase (Acc1) genes [24]. Furthermore, experimental studies demonstrated that estradiol reduced production of reactive oxygen species (ROS) and lipid peroxidation, thus inhibiting IkappaB-α degradation and nuclear factor-kappaB (NF-kB) activation [25]. With the aforementioned evidence, it was not surprising to see that the OVX rats in our study showed a higher degree of liver fat accumulation, inflammation and hepatocyte injury than in control rats.

High fructose consumption has been associated with increased de novo lipogenesis, insulin resistance and visceral adiposity in overweight/obese adults [26]. Animal studies showed that adding fructose in the diet significantly increased expression of lipogenic genes, such as Acc1, Fas and stearoyl CoA desaturase (SCD1) than high fat diet alone [27]. Moreover, fructose metabolites could directly activate transcriptional factors, such as sterol-regulatory element binding protein (SREBP)-1c and carbohydrate-response element binding protein (ChREBP), thus enhancing hepatic lipogenesis [27]. Fructose metabolism also leads to hepatic ATP depletion, formation of uric acid, ROS production and liver inflammation [28]. Similarly, our results showed that OVX + HFHF diet could induce a more severe form of NASH pathology than that seen in OVX alone. In line with histopathological changes, OVX + HFHF diet increased hepatic FFA contents, markers of lipid peroxidation (MDA) and inflammation (IL-6), and reduced hepatic levels of natural antioxidant (GSH).

Genistein has a similar structure to 17β-estradiol and can stimulate the transcriptional activity of estrogen receptor alpha and beta which makes it an attractive alternative to estrogen replacement therapy in post-menopausal women with NAFLD [29]. Independent of the estrogen effect, genistein could also induce the expression of peroxisome proliferators-activated receptor α (PPARα) which in turn regulates fatty acid β-oxidation pathways in the liver. The activation of PPARα prevents triglyceride accumulation and is associated with histological improvement of NASH in a human study [30, 31]. Our results conformed to other observation that genistein could reduce hepatic steatosis (as evidenced by decreased fat accumulation on Oil Red O stain and hepatic FFA contents), liver inflammation (both by histology and serum marker) and oxidative stress. Similar to our study, Susutlertpanya and colleagues demonstrated that genistein treatment decreased hepatic MDA and TNF-α levels and enhanced PPAR-ϒ expression along with the improvement in liver histology in rats with NASH. A recent study suggested that genistein might reduce inflammation in NASH through the reduction in endotoxin levels and toll-like receptor 4 gene expression [10].

Clinical and animal experiments showed that aerobic exercise was beneficial for NASH through several mechanisms [11]. Cho and colleagues demonstrated that treadmill running could significantly increase PPARα and carnitine palmitoyl transferase I (CPT-1) expression and decrease SREBP-1c, lipin1, and FAS expression leading to the improvement in NAFLD in C57BL/6 mice fed with high fat diet. They also showed that exercise enhanced the production of 5′ AMP-activated protein kinase (AMPK) leading to the increase in fatty acid β-oxidation and the attenuation of lipogenesis [32]. Furthermore, continuous running exercise has been shown to decrease lipid peroxidation and protein carbonylation in mouse liver [33]. In accordance with other studies, our results showed that running exercise could elicit the improvement in liver histology and the reduction in hepatic FFA and markers of inflammation (IL-6) and lipid peroxidation (MDA) in OVX + HFHF rats. We, however, did not evaluate the effect of exercise on insulin sensitivity in this study. Previous unpublished data from our group showed that insulin sensitivity did not change significantly in rats with NASH compared with control rats, possibly due to weight loss seen in our NASH model. Therefore, in the current study, we mainly focused on proving that this exercise protocol could reduce the severity of the NASH features by other mechanisms. Our results did show that it could improve NASH histopathology, probably by the reduction in inflammation and oxidative stress. Unfortunately, we cannot prove the association between this exercise protocol and insulin sensitivity. This is the limitation of our study and would evaluate this aspect in the next study.

In this study, neither genistein nor exercise could restore GSH levels in OVX + HFHF rats to the levels of a control group. A study by Wiegand et al. showed similar results in that genistein had no effects on hepatic enzyme activity of catalase (CAT), glutathione peroxidase (GPx) and superoxide dismutase (SOD) or glutathione levels [34], while an in vitro study demonstrated genistein increased the expression of GPx but not CAT or SOD. Data are conflicting regarding the effect of exercise on oxidative stress and it appears that the intensity and type of exercise may have different effects on GSH. Elokda and colleagues showed that combined aerobic and circuit weight training exercise increased GSH levels, while Ilhan et al. reported a more prominent increase in lipid peroxidation and a reduction in GSH in combined aerobic-anaerobic exercise group as compared to other types of exercise [35].

Interestingly, combining genistein and exercise did not provide an additional benefit on NAFLD compared with either treatment alone. Although there were no other studies that directly evaluated the effects of these treatment on NAFLD, a proteomics study using ovariectomized rats indicated that isoflavone and exercise combination therapy could favorably modulate hepatic protein expression toward normal values than either treatment alone [36]. Given that either treatment alone was so efficacious that it almost normalized histological and biochemical changes, we hypothesized that a small added benefit from combined treatment might not be apparent in this experimental study.

There were few limitations to our model. First, we used young OVX rats as a model for post-menopausal women. Applying these results to elderly women needed to take into consideration the effects of aging on treatment efficacy. Second, although our rats manifested on liver histology as NASH, they were lean because our model closely resembled the methionine-choline deficient diet model. This was in contrast with most patients with NASH who were obese. There might be differences in treatment efficacy between these two phenotypes.

## Conclusion

We found that estrogen deficiency induced by ovariectomy could lead to NAFLD development in this rat model with a more severe pathology when HFHF diet was added. Genistein and moderate intensity exercise could reduce fat accumulation, liver inflammation and oxidative stress, thus improving histological changes of NASH. Combining genistein and exercise did not provide additional benefits from either treatment alone. This is the first study to evaluation the combined effect of genistein and exercise in NASH. Clinical studies are warranted to confirm the beneficial effects of genistein in post-menopausal women with NAFLD.

## Supplementary information

**Additional file 1: Supplement Figure 1.** Chemical structure of genistein (C_15_H_10_O_5_).

## Data Availability

Materials described in the manuscript, including all relevant raw data, will be freely available to any scientist wishing to use them for non-commercial purposes upon request.

## Abbreviations

NAFLD: Nonalcoholic fatty liver disease
NASH: Nonalcoholic steatohepatitis
OVX: Ovariectomized
HFHF: High fat high fructose
FFA: Free fatty acid
IL-6: Interleukin-6
MDA: Malondialdehyde
GSH: Glutathione
ELISA: Enzyme-linked immunosorbent assay
DMSO: Dimethyl sulfoxide
