# Supplementary figures and images for: Genistein and exercise modulated lipid peroxidation and improved steatohepatitis in ovariectomized rats
Source: BMC Complement Med Ther. 2020 Jun 1;20:162. doi: 10.1186/s12906-020-02962-z (PMC7262771; doi:10.1186/s12906-020-02962-z)

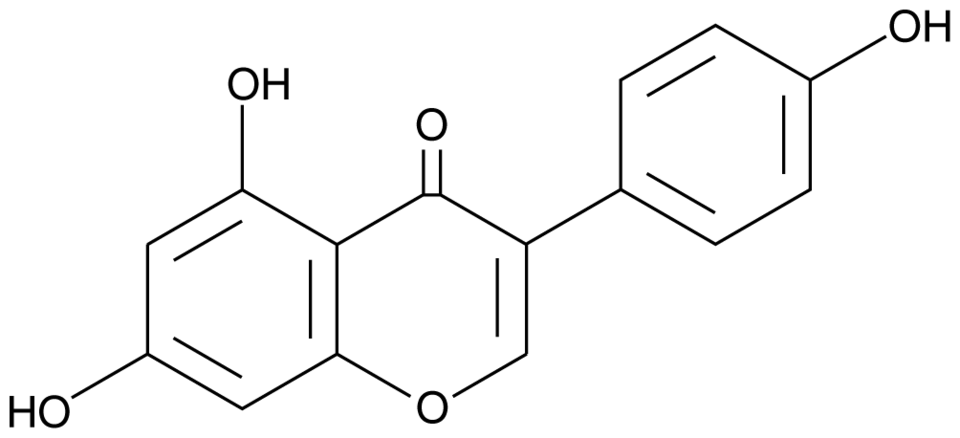


**Supplement figure 1. Chemical structure of genistein (C15H10O5)**

Supplement: Supplementary file 1 — Additional file 1: Supplement Figure 1. Chemical structure of genistein (C15H10O5). [file 12906_2020_2962_MOESM1_ESM.docx]
